# Using inverted autogenous veins to substitute arteries in a canine model

**DOI:** 10.4103/0974-2700.66523

**Published:** 2010

**Authors:** Saman Nikeghbalian, Seyed Mohammad Vahid Hosseini, Ali Mohammad Bananzadeh, Ahmad Monabati, Mohammad Hadi Bagheri, Tannaz Razmi, Seyed Ali Malek-Hosseini

**Affiliations:** Department of Surgery, Shiraz University of Medical Sciences, Shiraz, Iran

**Keywords:** Arterial anastomosis, inverted autogenous graft, vascular conduit

## Abstract

**Aims:**

Rapid harvesting of autogenous graft over a wire is an optional way in trauma surgery and it places the inverted conduit so that its adventitial layer is within the lumen of the graft. Our aim of this study was to compare the patency of inverted autogenous graft vs noninverted graft in dogs.

**Settings and Design:**

Experimental animal models.

**Materials and Methods:**

In this experimental study, 12 dogs were anesthetized and 10 cm of the external jugular vein was excised. The vein was equally divided into two 5-cm sections. One section was inverted and the other was left intact. Afterward, 5 cm of both the femoral arteries were removed and the right (inverted) and the left (not inverted) arteries were grafted, respectively. The patency of the arteries was evaluated by Color Doppler ultrasonography immediately postoperation and up to 6 months thereafter.

**Statistical Analysis:**

Data were analyzed with Fisher's exact test using SPSS version 15. *P* value below 0.05 was significant.

**Results:**

None of the 12 inverted vein grafts were patent at 3^rd^ to 14^th^ days follow-up with Doppler ultrasonography. All of them were completely obstructed by thrombosis. However, 11 (92%) of the noninverted vein grafts were patent both at 3 and 6 months follow-up. One of the noninverted grafts was almost completely obstructed with thrombosis (90%) and the other 2 were incompletely obstructed with intimal thickening.

**Conclusions:**

Despite many favorable results in the previous studies with regard to excellent patency of inverted vein graft, our results were disappointing and we recommend using the graft in the right direction and taking care to preserve the intima intact.

## INTRODUCTION

Autogenous vein graft has been widely used in vascular surgery, especially if it can be harvested rapidly and it seems to be an ideal graft for the small caliber vessels and trauma surgery. However, long-term observation revealed some disadvantages, such as size discrepancy, intimal thickening, intimal hyperplasia, thickening of the venous valves, and aneurismal dilatation.[1] Additionally, it is usually abandoned in the case of a thrombosed or phlebitic vein. Recently, synthetic grafts have been used more frequently. However, these materials have been shown to have multiple limitations when used in small caliber vessels; therefore, recent experimental studies have considered inverted autogenous vein graft as a good substitute and they can be harvested over a wire very rapidly in emergency situations. The strong adventitial layer not only prevents aneurismal dilatation but also has a good antithrombotic activity as reported in many studies.[1–5]

Our aim of this study was to compare the patency of inverted autogenous grafts vs noninverted grafts in an experimental animal study.

## MATERIALS AND METHODS

An animal model study was conducted on 12 dogs weighing 10–18 kg, and the protocol was approved by the ethics committee of the Shiraz University of Medical Sciences.

They were intubated and connected to the respirator and operated in sterile condition under general anesthesia (ketamine 50 mg/kg). Ten centimeters of the external jugular vein was removed for use as a graft. The vein was divided into 2 equal segments. The adipose tissue was removed carefully from the surface of the adventitia in half the segments and they were inverted, whereas the other segments were left intact. The inverted and noninverted segments were kept in separate heparin saline solutions at room temperature and proximal and distal ends were irrigated with heparin saline.

Through bilateral inguinal incisions, approximately 5 cm of the left and right femoral arteries were removed immediately following the autogenous vein graft harvesting. The inverted veins were anastomosed to the right femoral arteries using 6-0 Prolene suture (continuous sutures, both-ends spatulated, with good growth factor) in an end-to-end fashion, but whereas the left sides were anastomosed with intact graft in the same way as in the right sides. All anastomoses were completed within 2 h after removal of the vein grafts.[1]

Postoperative anticoagulant therapy was not applied and antibiotics were given for 3 days. Patency of the grafts was evaluated by Color Doppler Ultrasonography (Schiller AG, Germany) with 4 and 10 MHz probes, in which the presence of flow was evaluated. It was performed immediately postoperatively and daily for 2 weeks, then monthly up to 6 months thereafter under ketamine anesthesia.[1]

After complete thrombosis of each conduit, it was resected during postoperative period and the normal side was followed-up to 6 months when the animals were sacrificed and the specimens were examined by gross inspection, light microscopy, and histopathologic assessments for chronic changes. All the specimens were placed in buffered formalin after removal. They were processed after 24 h for paraffin embedding. A fire microthick slide was prepared from each block and stained by hematoxylin and eosin and examined under a light microscope.

### Data analysis

The data were analyzed with Fisher's exact test using SPSS version 15 (Ballynahinch / Ireland). P value below 0.05 was significant.

## RESULTS

The study on all the dogs was completed, and none of them had any wound infection. On the right side, the length of the jugular vein ranged from 3.2 to 4.2 cm, whereas on the left side, the length ranged from 3.3 to 4 cm. The diameter of the grafts ranged from 8 to 12 mm on both the sides. The diameter of femoral arteries ranged from 5 to 8 mm on both the sides.

### Doppler evaluation

After the 14^th^ day of observation, none of the inverted vein grafts were patent. However, 11 of the 12 (92%) anastomosed by conventional method were patent during the monthly follow-up with Doppler ultrasonography (*P* < 0.0001) [Table 1].

**Table 1:** Results of the experimental study

No. of the dog	Doppler‡		Pathologic study		Graft	
	Rt†	Lt	Rt	Lt	Length (Rt/Lt) (cm)	Diameter (Rt/Lt) (mm)
1	Occluded	Patent	Thrombosed	50% Occluded	3.5/3.5	8/9
2	Occluded	Patent	Thrombosed	Thrombosed	4/3.5	9/9
2	Occluded	Patent	Thrombosed	Thrombosed	4/3.5	9/9
3	Occluded	Patent	Thrombosed	Normal	3.2/3.3	10/12
4	Occluded	Patent	Thrombosed	Normal	4/4	12/12
5	Occluded	Patent	Thrombosed	Normal	4.2/4	12/12
6	Occluded	Patent	Thrombosed	Normal	3.5/4	11/11
7	Occluded	Patent	Thrombosed	Normal	4/4	10/11
8	Occluded	Patent	Thrombosed	50% Occluded	3.5/4	11/11
9	Occluded	Patent	Thrombosed	Normal	4/4	12/11
10	Occluded	Patent	Thrombosed	Normal	4/4	8/8
11	Occluded	Patent	Thrombosed	Normal	3.5/3.3	9/9
12	Occluded	Patent	Thrombosed	Normal	3.2/3.4	9/10

† ON THE RIGHT SIDE, THE INVERTED VEIN GRAFT WAS USED AND ON THE LEFT SIDE A SIMPLE NONINVERTED VEIN GRAFT

‡ DOPPLER RESULTS DURING 6 MONTHS FOLLOW-UP

### Pathologic evaluation

**Figure 1:**
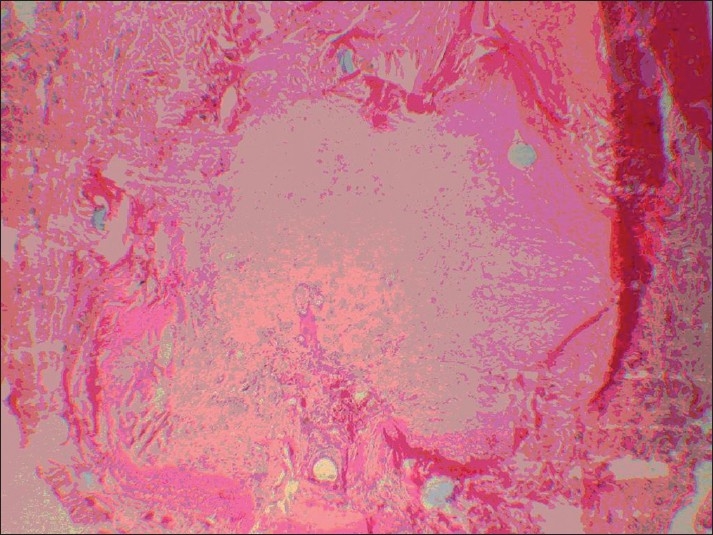
Strand of fibrous tissue

On the right side (inverted vein graft), all the samples were completely thrombosed, which started from the 3^rd^ to 14^th^ day. The anastomosis was completely occluded and filled with a large number of fibroblasts. No epithelialized canal at the site of anastomosis was observed and only a strand of fibrous tissue was present [Figure 1].

**Figure 2:**
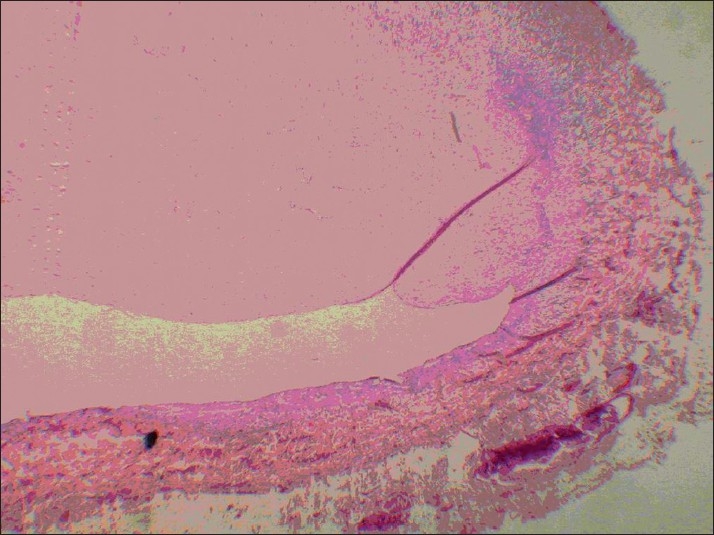
Thrombosis and intimal hyperplasia

**Figure 3:**
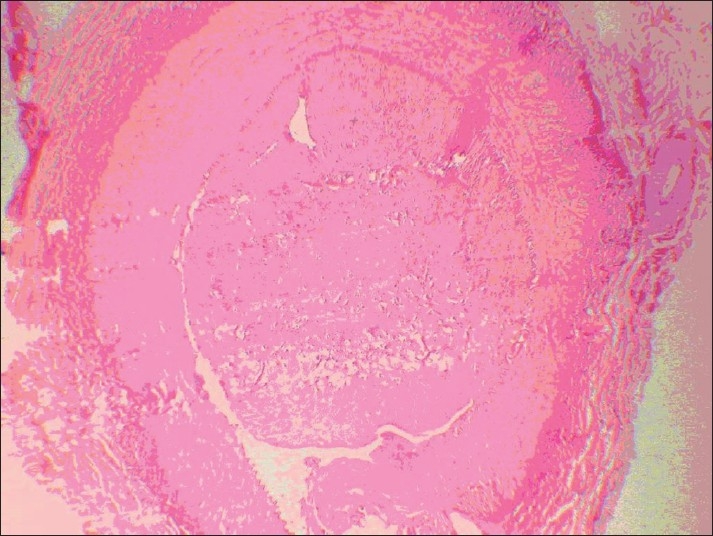
Organization of thrombosis

**Figure 4:**
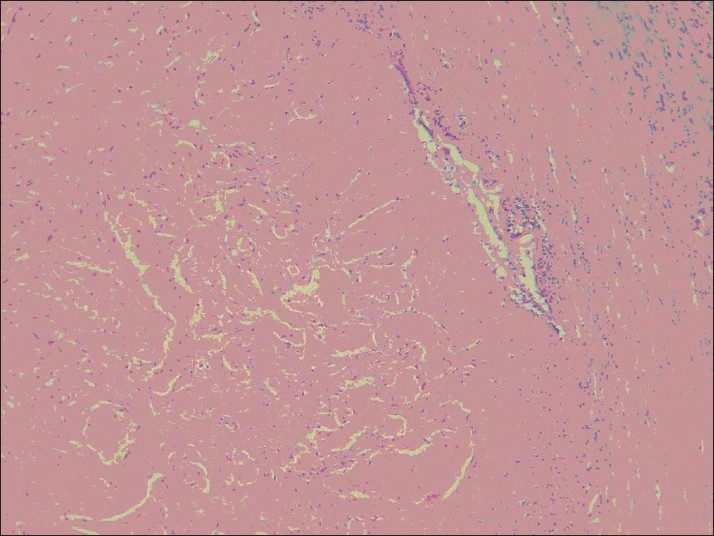
Recanalization of thrombi was evident

**Figure 5:**
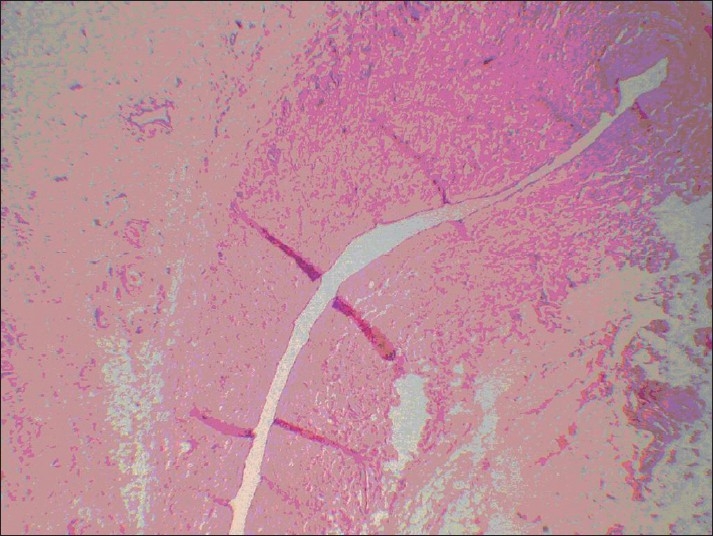
Thin and regular

**Figure 6:**
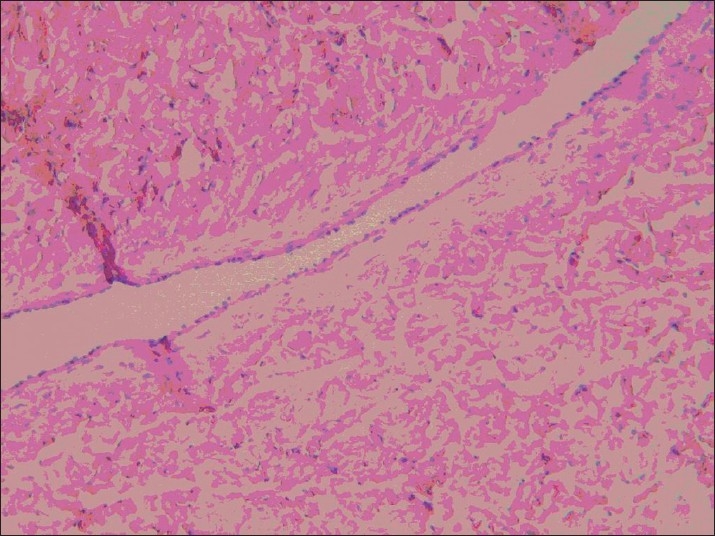
External elastic membranes were easily detectable

However, on the left side (conventional vein graft), only 1 case was completely obstructed with thrombosis (8.3%), and in 2 other cases the lumen was partially (50% of the lumen) obstructed with thrombosis and intimal hyperplasia (*P* < 0.0001) [Figure 2]. The thrombosis in the completely obstructed vein was of the old type, with organization of thrombosis [Figure 3]. Recanalization of thrombi was evident [Figure 4]. The other 9 samples (75%) were completely open, and normal amount of intimal thickening was observed. The walls were thin and regular [Figures 5 and 6]. External elastic membranes were easily detectable [Figure 6].

## DISCUSSION

Autogenous vein graft was first applied clinically as an arterial substitute by Jose Goyanes in 1906 and Erich Lexer in 1907 independently.[6] The experimental or clinical studies have shown several disadvantages of autogenous vein grafting, such as initial thrombosis, intimal thickening, aneurismal dilatation, or valvular hypertrophy. Also it is well known that synthetic grafts have a limitation for use in the small caliber vessels, such as the superficial femoral, renal, or coronary arteries, but during the early years of vascular prosthetic development, the characteristics of the *ideal graft* had not been defined and many experiments were performed to introduce an acceptable vascular conduit.[7]

Moseley *el al*.[8] reported that the surface of the endarterectomized arteries was covered by neoendothelial cells in the following month after surgery. Iwai *el al*.[1] investigated the efficacy of inverted autogenous vein graft in both humans and canines. Because the adventitial layer is strong and has good fibrinolytic activity, it may prevent aneurismal formation in an autogenous graft.[1,4,5] They used inverted vein grafts in 9 patients and on 25 mongrel dogs; and of the 9 patients, 5 had arteriovenous shunting for hemodialysis, 1 had replacement of femoral artery, and 3 had inverted vein patch to the iliac or femoral arteries. They followed the patients from 1 month to 1 year with good patency.[1] In our study, we observed that all the inverted vein grafts failed and the grafts were occluded by thrombosis through the 3^rd^ to the 14^th^ day of follow-up.

In another study performed by Szilagyi *el al*., the long-term patency rate of noninverted vein graft in femoropopliteal bypass was evaluated. They reported a 64% patency rate after 5 years and 44% after 10 years. The rest of the cases developed progressive stenosis or dilatation of the graft wall.[9–11] We also obtained a 92% patency rate after 6 months of follow-up.

Todd *el al*.[12] studied the fibrinolytic activities in harvested graft and have reported that reduction of fibrinolytic activity started in the intima and finally reached adventitia, and so if endarterectomized arteries with exposed subendothelial collagen could remain patent, the inverted graft theoretically would have had better survival than the rough endarterectomized surface. However, in our pathologic results from inverted graft, the anastomosis was completely occluded and filled with a large number of fibroblasts. No epithelialized canal at the site of anastomosis was observed, and only a strand of fibrous tissue was present. This meant that fibrinolytic activities of the adventitial layer are not adequate for the prevention of diffuse thrombosis that has been activated by contact with this layer.

Iwai *el al*.[1] also described the important role of preparation media and temperature in diminishing the time of fibrinolytic activator. We also kept the sample in solution and temperature as done by Iwai *el al*. and tried to perform the anastomosis within 2 h after harvest.

Stenosis increases the incidence of thrombosis and occlusion of the graft. In our study, the internal diameter of the jugular vein grafts used a range of 8–12 mm. On the other hand, the diameters of the femoral arteries were between 5 and 8 mm and we used continuous sutures but we kept adequate growth factor in anastomosis. Therefore, the thrombosis in the inverted veins could not be attributed mainly to stenosis.

In humans, the spread of the endothelial pannus that originates at the anastomoses is very limited when compared with healing in various other animals in which it occurs rapidly and may be complete in 4–8 weeks.[8,11] The behavior of prostheses in dogs most closely parallels that in humans, thus explaining the emergence of the dog as the favorite animal model for studying vascular grafts.

We encountered thrombosis in all our inverted grafts, which is against Iwai *el al*.’s[1] results. The technical problems usually occur during the first 24 h but those related to the nature of the graft appear later, as seen in our study. Our results of the noninverted grafts with the same technique of anastomosis and preparation are similar to those of other recent studies.[11] Our control of noninverted graft rules out the technical errors and recommends further clinical trials and multicenter studies for the evaluation and safety of these kind of grafts in humans.

The clinical trial is defined as any study that prospectively assigns human subjects to either an intervention or comparison group to evaluate the causal relationship between a medical intervention and outcome and should be approved by ethical committee and with patients' consent; however, in animal models also the ethical aspect should be considered.[13]

Saphenous graft is one the autogenous grafts that has been mostly used in vascular surgery not only in elective procedure but widely in emergency situations; however, the much felt need for rapid harvesting of graft has led to efforts to come up with techniques, such as over-the-wire saphenectomy. This technique is rapid and simple because through a small stab incision, the saphenectomy wire is entered and depending on the length required, it is brought out through another small incision.[5] More autogenous grafts with better prolonged patency will be available if harvested with great care and used in the right direction.[7] Many studies[1] reported the good patency of inverted autogenous grafts that can be harvested over the wire rapidly, but the results of our studies differed from theirs and emphasized the importance of an intact intima in the prolongation of graft patency.

## CONCLUSION

We still recommend that autogenous graft be harvested with care for the intimal layer and in the right direction to get better results for early and long patency.
